# Prevalence and burden of gastrointestinal parasites of Djallonke sheep in Ayeduase, Kumasi, Ghana

**DOI:** 10.14202/vetworld.2016.361-364

**Published:** 2016-04-09

**Authors:** Moses Owusu, Jemima Owusu Sekyere, Frederick Adzitey

**Affiliations:** 1 Department of Pathobiology, School of Veterinary Medicine, College of Health Sciences, Kwame Nkrumah University of Science and Technology, Kumasi, Ghana; 2 Department of Nursing, Signature Healthcare of Madison, Madison, Tennessee, United States of America; 3 Department of Animal Science, Faculty of Agriculture, University for Development Studies, Tamale, Ghana

**Keywords:** burden, Djallonke sheep, gastrointestinal parasites, prevalence

## Abstract

**Aim:**

This study was conducted to determine the prevalence and burden of gastrointestinal (GIT) parasites of Djallonke sheep in Ayeduase, Kumasi from January 2015 to July 2015.

**Materials and Methods:**

The presence of nematodal eggs and coccidial oocysts in fecal samples were analyzed using the saturated sodium chloride floatation technique. Identification of eggs or oocysts was done on the basis of morphology and size of the eggs or oocysts.

**Results:**

Out of 110 fecal samples of sheep examined, 108 were infected with GIT parasites, representing a prevalence rate of 98.2%. The total infection rate of GIT nematodes and coccidia oocysts were 94.5% and 51.8%, respectively. Strongyle nematode (94.5%) was the most prevalent GIT nematode detected, followed by strongyloides (27.3%). The average nematodal burden in g/feces was significantly higher (p<0.001) in young rams under 1 year (3482.0) than gimmers (1539.0), lamb (825.0), ewes (420.7), and rams over 1 year (313.3). Nematodal burden in gimmers was significantly higher (p<0.001) than that of lambs, ewes, and rams over 1 year. Nematodal counts of lambs, ewes, and rams did not differ significantly (p>0.05) from each other. The average coccidia oocysts count in g/feces was significantly higher (p<0.001) in lambs (2475.0) than rams under 1 year (286.0), gimmers (263.6), ewes (158.6), and rams over 1 year (150.0). There was no significant difference (p>0.05) in the coccidia oocysts count of rams under 1 year, gimmers, ewes, and rams over 1 year. From the studied animals, 40%, 6.36%, 48.18%, and 5.45% had heavy, moderate, light, and no infestation, respectively, with GIT nematodes.

**Conclusion:**

Djallonke sheep in Ayeduase, Kumasi, were infested with varying amounts of GIT parasites. The infestation of Djallonke sheep by GIT parasites also varies among different age groups and sexes.

## Introduction

The rich potential from the small ruminant sector is not efficiently exploited due to several constraints including malnutrition, inefficient management, and diseases [1,2]. In this regard, diseases due to parasites take the lion’s share in limiting the productivity of these animals all over the world. This is especially truein many tropical and subtropical regions. Small ruminants under intensive and extensive production systems are extremely susceptible to the effects of a wide range of helminths [2]. The prevalence of gastrointestinal (GIT) helminths is related to the agro-climatic conditions such as quantity and quality of pasture, temperature, humidity, and grazing behavior of the host [3].

GIT parasite infections are a world-wide problem for both small- and large-scale farmers, but the impact in general is greater in Sub-Saharan Africa [2]. Among the parasitic diseases, endoparasites are of greatest importance in sheep and goats. Common parasites of sheep and goats include coccidia, roundworms, tapeworms, and liver flukes [4]. Strongyle nematodes are the main cause of parasitic gastroenteritis in sheep and goats in Ghana [5]. Young animals and those with weakened immune systems due to other diseases are most affected by internal parasites [6].

Economic losses are caused by GIT parasites in a variety of ways: They cause losses through lowered fertility, reduced work capacity, involuntary culling, a reduction in food intake and lower weight gains, lower milk production, treatment costs, and mortality in heavily parasitized animals [7]. A combination of treatment and management is necessary to control parasites so that they will not cause economic loss to the producer [6]. Internal parasites get out of control and cause damage when their numbers grow beyond what the animal can tolerate.

It is, therefore, necessary to carry out a study to determine the prevalence and burden of GIT parasites in sheep. This will determine whether there is the need to develop preventive and treatment measures to curb production losses. The objective of this work was to determine the prevalence and burden of GIT parasites in Djallonke sheep in Ayeduase.

## Materials and Methods

### Ethical approval

The research was conducted after approval of research committee and institutional ethics committee.

### Study area

The study was conducted in Ayeduase in the Kumasi Metropolis. The metropolis is located in the north-eastern part of the Ashanti Region of the Republic of Ghana between latitude 6°35’’ and 6°40’’ N and longitude 1°30’’ and 1°35’’ E [8]. During the study period, Ayeduase received an average annual rainfall of 1100-1600 mm and a mean monthly temperature of 33.33-34.0°C [8]. The vegetation of the metropolis falls within the moist semi-deciduous section of the South-East Ecological zone [8].

### Collection and examination of fecal samples

A total of 110 rectal fecal samples from apparently healthy sheep were collected from 12 different flocks in Ayeduase. Fecal samples were placed in screw-capped plastic bottles, labeled and immediately transported under 4°C to the Ashanti Regional Veterinary Diagnostic Laboratory in Kumasi for analyzes. The category of sheep and the number of samples examined are presented in Table-1. The numbers examined varied because the number and ages of sheep in a farmer’s flock vary.

**Table-1:** Category of sheep and number of samples examined in Ayeduase.

Category of sheep	Number of samples analyzed
Rams above 1 year	15
Rams below 1 year	25
Ewes	29
Gimmers	33
Lambs	8
Total	110

The presence of nematodal eggs and coccidial oocysts in all fecal samples was analyzed using the saturated sodium chloride floatation technique [9-11]. Identification of eggs or oocysts was done on the basis of morphology and size of the eggs or oocysts [10]. In this study, the authors wished to determine species, but financial constraints and lack of some equipment/instruments in our laboratory did not permit that. Fecal egg counts per gram were determined for each sample following the McMaster egg counting technique, and the degree of infection was categorized based on Hansen and Perry [10]. Animals with egg counts from 50-799, 800-1200, and above 1200 were categorized as lightly, moderately, and heavily infected, respectively [9-11].

### Data analysis

Data obtained was analyzed using analysis of variance of the Genstat Edition four.

## Results and Discussion

The results for the prevalence of GIT parasites in sheep are presented in Table-2. Microscopic examination of the 110 sheep fecal samples revealed that 98.2% (108) of sheep were infested with one or more GIT parasites. Emiru *et al*. [12], Admasu and Nurlign [13], and Gadahi *et al*. [14] also found that 84.3%, 58.7%, and 53.3% of sheep fecal samples, respectively, were infested with one or more GIT parasites. The total infestation rate of GIT nematodes was 94.5% (104) while that of coccidia was 51.8% (57). Similarly, to this study Aragaw and Gebreegziabher [15] reported an overall prevalence rate of nematodes in sheep to be 93.1%. Wang *et al*. [16] found an overall prevalence of coccidial infection in sheep to be 92.9% (287/309) which is higher than what was found in this study. Out of 104 samples that were infested with nematodes 51.1% (53/104) of them were also infested with coccidia.

**Table-2:** Prevalence of gastrointestinal parasites of sheep in Ayeduase.

Category Sheep	Number of samples examined	Number of samples infected with one or more GIT parasites (%)	Number of samples infected with nematodes (%)	Number of samples infected with coccidiaoocysts (%)
Rams over 1 year	15	15 (100)	13 (86.7)	9 (60)
Rams below 1 year	25	25 (100)	25 (100)	14 (56)
Ewes	29	28 (96.6)	27 (93.1)	13 (44.8)
Gimmers	33	33 (100)	33 (100)	14 (42.4)
Lambs	8	7 (87.5)	6 (75)	7 (87.5)
Total	110	108 (98.2)	104 (94.5)	57 (51.8)

GIT=Gastrointestinal

The nematodes detected were the strongyle type (104/110) and *Strongyloides* spp. (30/110); and thus, the strongyle type nematodes were the most prevalent GIT nematodes detected. Furthermore, all the samples infested with strongyloides were also infested with strongyle. Nematode parasites (54.1%), *Eimeria* (14.6%), and *Moniezia* (13.4%) were also found to infest small ruminants [12].

The prevalence of nematodes in sheep was highest in gimmers (100%) and rams below 1 year (100%), followed by ewes (93.1%), rams over 1 year (86.7%), and lambs (75%). This means that more gimmers and rams below 1 year were infested with GIT nematodes, followed by ewes, rams over 1 year and lambs. The low prevalence of nematodes in lambs could be because, in some flocks, sampled lambs had just started nibbling and may not have ingested contaminated feed that leads to GIT parasites infestation. Quantitative analyzes of sheep fecal samples showed that the nematodal burden of rams under 1 year was significantly higher (p<0.001) than gimmers, lambs, ewes, and rams over 1 year (Table-3). Quantitatively, the number of gimmers infested with nematodes was also significantly higher (p<0.001) than that of lambs, ewes, and rams over 1 year. Thus, the total number of nematodes isolated from rams under 1 year was the highest, followed by those isolated from gimmers, lambs, ewes, and rams over 1 year.

**Table-3:** Prevalence and burden of nematodes ova and coccidia oocysts of sheep in Ayeduase.

Parasite type	Sheep type						

	Ewes	Lambs	Gimmers	Ram+	Ram−	LSD*	p value
Nematodes eggs/g of feces	420.7^a^	825^a^	1539.4^b^	313.3^a^	3482^c^	810.8	<0.001
Coccidiaoocysts/g of feces	158.6^a^	2475^b^	263.6^a^	150^a^	286^a^	747.4	<0.001

Ram+=Rams over 1 year, Ram−=Rams under 1 year, LSD=Least significant difference, p value=Probability value, means in the same row with different superscript are significantly different (p<0.05)

The prevalence of coccidia oocysts in sheep was highest in lambs (87.5%), followed by rams over 1 year (60%), rams below 1 year (56%), ewes (44.82%), and gimmers (42.4%) (Table-2). Quantitative analyzes of sheep fecal samples showed that the coccidial burden in lambs was significantly higher (p<0.001) than rams under 1 year, gimmers, ewes, and rams over 1 year. Contrarily, to the results obtained for nematodal burden, lambs were most infested with coccidia oocysts both qualitatively and quantitatively. In agreement with this study Emiru *et al*. [12] found significant differences (p<0.05) in the prevalence of GIT parasites between the different age groups. Young, unhealthy, and malnourished sheep will be more susceptible to nematode and coccidia parasites. Sheep are infested with nematode and coccidia parasites when they eat feed or drink water contaminated with the eggs of these parasites.

The intensity of infestation of GIT nematodes in sheep in Ayeduase is shown in Table-4. Out of the total sheep fecal samples (110) examined, 40% (44), 6.4% (7), and 48.2% (53) had heavy, moderate, and light infestations, respectively, with GIT nematodes, while no GIT nematode ova was detected in fecal samples from 5.5% (6) of the study population. Admasu and Nurlign [13] reported that 91 (58.7%), 34 (21.9%), and 30 (19.4%) of sheep were lightly, moderately, and highly infested by GIT parasites.

**Table-4:** Intensity of gastrointestinal nematodes infestation of sheep in Ayeduase.

Category sheep	Heavy (%)	Moderate (%)	Light (%)	No infestation (%)
Rams over 1 year	-	-	13 (86.7)	2 (13.3)
Rams below 1 year	25 (100)	-	-	-
Ewes		2 (6.9)	25 (86.2)	2 (6.9)
Gimmers	19 (57.6)	5 (15.2)	9 (27.3)	-
Lambs	-	-	6 (75)	2 (25)
Infestation intensity (%)	44 (40)	7 (6.4)	53 (48.2)	6 (5.5)

The prevalence of GIT parasites by sex is shown in Table-5. Female sheep (98.6%) and male sheep (97.6%) were infested with one or more GIT parasites. More female sheep 66 (95.7%) were infested with nematodes than male sheep 38 (92.7%). With regards to coccidia oocysts, more male sheep 23 (57.5%) were infested than female sheep 34 (49.3%). Admasu and Nurlign [13] found no significant differences (p>0.05) in the prevalence of GIT parasites between sex, while Emiru *et al*. [12] found significant differences (p<0.05) in the prevalence of GIT parasites between sex.

**Table-5:** Prevalence of gastrointestinal parasites by sex.

Sex	Number of samples examined	Number of samples infected with one or more GIT parasites (%)	Number of samples infected with nematodes (%)	Number of samples infected with coccidiaoocysts (%)
Male	41	40 (97.6)	38 (92.7)	23 (57.5)
Female	69	68 (98.6)	66 (95.7)	34 (49.3)
Total	110	108 (98.2)	104 (94.5)	57 (51.8)

GIT=Gastrointestinal

## Conclusion

Djallonke sheep in Ayeduase are infested with nematodal and coccidial parasites. The prevalence rate of these GIT parasites varies among different age groups and sexes. The intensity of infestation by GIT nematodes also varies from no to heavy infestation. There is the need for farmers in Ayeduase to improve on their biosecurity and feeding habits to prevent GIT parasite infestations in their flocks. Sheep should be dewormed during the dry season (December to March) to reduce the shedding of ova and larvae and pasture contamination during the subsequent rains. They should be dewormed again during the peak rainy season (June-July) to prevent massive pasture contamination with ova and larvae from young adults developed from infections picked up during the early part of the rains.

## Authors’ Contributions

FA supervised the overall research work. MO and JOS went to the field to collect the data and carried out the research. MO and FA wrote the first draft before being revised by all the authors. All authors also read and approved the final manuscript.
